# The Spinal Facilitation Hypothesis and Reflex Arcs in Modern Osteopathic Medicine

**DOI:** 10.7759/cureus.91782

**Published:** 2025-09-07

**Authors:** Bruno Bordoni, Marta Simonelli, Bruno Morabito, Allan R Escher

**Affiliations:** 1 Physical Medicine and Rehabilitation, Foundation Don Carlo Gnocchi, Milan, ITA; 2 Neuroscience, Mental Health and Sensory Organs (NESMOS), Sapienza University of Rome, Roma, ITA; 3 Physical Medicine and Rehabilitation, Fondazione Policlinico Universitario Agostino Gemelli, Università Cattolica Del Sacro Cuore, Roma, ITA; 4 Oncologic Sciences, University of South Florida Morsani College of Medicine, Tampa, USA; 5 Anesthesiology/Pain Medicine, H. Lee Moffitt Cancer Center and Research Institute, Tampa, USA

**Keywords:** manual therapy, osteopathic manipulative medicine, osteopathy, pain, spinal cord

## Abstract

The spinal cord is an organ capable of sending and receiving a lot of biological and electrical information. It is not just a sending and receiving channel, but a living structure capable of autonomously processing the afferent and efferent notifications with which it comes into contact. The osteopathic neurological model includes the concept of facilitation of the spinal segment, that is, a reflex arc that is established in a spinal segment between two visceral and/or somatic structures, creating a loop of chronicity. This mechanism that implies a reflex arc would explain the somatic symptoms coming from a visceral disorder, or vice versa, involving the specific neural segment of visceral and somatic innervation. Such a reflex arc, due to the bioelectrical complexity of the nervous system, may not occur. The article briefly reviews some concepts of neurophysiology to raise new reflections on whether or not to consider the hypothesis of the facilitated segment and to have a different vision in clinical pictures that are not always easy to frame.

## Introduction and background

The founder of Osteopathic Manipulative Medicine (OMM), Andrew Taylor Still, (1874), coined the term “osteopathic lesion” [1]. With the term “lesion,” he meant a dysfunction of the symptomatic area, with interruption of fluid flow. Still and his students considered the human body as a functional unit, associating the osteopathic lesion not only arising from the somatic system but also in conjunction and/or as a contributory cause with the visceral system [2]. Guy Dudley Hulett gave an early classification of osteopathic lesions, such as complete shift, partial shift, and visceral displacement [1]. Hulett proposed the concept that, in addition to the lesions described, there were concomitant small somatic lesions at the spinal level. George Malcom McCole proposed in 1935 new classifications of osteopathic lesions, such as traumatic (lesions capable of negatively reflecting on the spinal column), reflectory (symptom different from the site of the cause with spinal stimulation of the adjacent segment), acute (traumatic or reflectory and can evolve from chronicity), and chronic lesions (traumatic or reflectory) [1]. Yale Castlio began to propose differences in the definition of osteopathic lesions (functional limitation) and spinal lesions (limitation of movement between the vertebral joints); the former could occur in an area distant from the origin of the symptom, involving somatic and visceral structures, and the latter could involve nervous structures such as the sympathetic ganglia. Castlio put forward an innovative concept, that is, a lesion that arises from an area distinct from the symptom can lead to tissue dysfunctions that involve the lesion and the entire organism (over time) [1].

John Martin Littlejohn gave an interpretation of lesion starting from a different reflection of the human body: no longer a machine, but an organism. According to the osteopathic lesion concept, the latter can negatively impact physical and cognitive health, introducing the term "environmental lesion" [1]. Harrison Fryette defined the term “total lesion,” a dysfunction that can also derive from eating behavior and pathogenic agents [1].

George MacDonald and W. Hargrave-Wilson wanted to make a distinction between a primary osteopathic lesion and a secondary or reflectory one; the first derives from an acute biomechanical lesion involving a joint, while the second can arise from causes that are not only mechanical but also from altered emotional status, which causes the primary lesion to persist [1]. Secondary lesions are activated by vascular, endocrine, and neurological reflex arcs, the latter of which can be stimulated by an irritated area in the spinal cord, involving not only the somatic area but also the visceral one.

In the 1960s, Ira Rumney changed the term “osteopathic lesion” to “somatic dysfunction” to arrive at the latest definition dictated by the Educational Council on Osteopathic Principles (ECOP) (Glossary of Osteopathic Terminology) of 2017 [3-5]. ECOP defined a somatic dysfunction as follows: “Impaired or altered function of related components of the body framework system: skeletal, arthrodial, and myofascial structures, and their related vascular, lymphatic, and neural elements.” [5]. ECOP also distinguishes between an acute and a chronic somatic dysfunction.

To diagnose the presence of a somatic dysfunction, the clinician can use TART (texture abnormality, asymmetry, restriction of motion, and tenderness), tissue alterations that can be found on palpation and that must be present in the area evaluated (one or all four criteria) [6]. Somatic dysfunction is related not only to a visceral problem but also to psychological sphere such as depression [7].

A theory that has guided many osteopathic clinicians to understand somatic dysfunction/lesion was devised by Denslow and Korr, calling it “spinal facilitation” [8,9]. The latter is seen as a chronic dysfunction of the spinal cord level causing dysfunctions related to the afferent/efferent area of the specific dorsal horn, with negative central influences as well [8]. Other authors supported this theory over time, proposing different related mechanisms, such as Wilbur Cole, Michael M. Patterson, and Richard L. Van Buskirk [1].

Another historical figure in osteopathic medicine is Louisa Burns, who, despite her motor disability due to a previous meningitis, was a pioneer in experimental research [10]. The concepts of viscerosomatic and somatovisceral reflex arcs derive from her experiments on animal models [11]. She demonstrated the presence of reflexes between the visceral and somatic areas.

Generally, these reflex arcs and dysfunctions are represented as a segmental spinal alteration, with aberration of the perception of sensitivity (afferences), of the resulting responses (efferents), and of the ascending/descending spinal pathways in a very simplistic way [2]. The reality of living is much more complex. The article briefly reviews some concepts of neurophysiology to raise new reflections on whether or not to consider the hypothesis of the facilitated segment and to have a different vision in clinical pictures that are not always easy to frame.

## Review

Facilitated segment

We can find different descriptions of reflex arcs in osteopathic medicine, defined as somatosomatic, somatovisceral, viscerosomatic reflex arcs. From ECOP, the first reflex is described as “localized somatic stimuli producing patterns of reflex response in segmentally related somatic structures”. The second arc is defined as “localized somatic stimuli producing patterns of reflex response in segmentally related visceral structures.” Finally, the last viscerosomatic reflex is defined as “localized visceral stimuli producing patterns of reflex response in segmentally related somatic structures” [5].

The viscerosomatic reflex is a disorder that arises from visceral functional alterations, which manifest themselves as a somatic dysfunction [2]. This disorder should be palpated at the level of the paraspinal musculature of the facilitated spinal segment. Visceral afferents derive from structures related to the viscera, its movements (expansions and contractions), the contents of the organs themselves; inflammation, ischemia; and information from the thoracic viscera and those located in the abdomen travel to the central nervous system (brainstem) via the parasympathetic pathways (vagus nerve to the nodose ganglion), entering the spinal cord via the dorsal root ganglia (DRGs) [12]. Vagal neurons carry information related to visceral function and can induce sensations such as satiety, dyspnea, and cough; spinal neurons can carry nociceptive sensations [12]. The enteric nervous system projects information to the efferent sympathetic ganglia [12]. All afferent pathways can carry pathognomonic symptoms. Visceral afferents (these pathways accompany sympathetic efferent fibers) can cause adaptations in the short and long term; if the stimulation has a limited duration, this will lead to a visceral reflex, while if the visceral information is long-lasting and/or intense, the central nervous system will be involved, also involving somatic responses (viscerosomatic reflex) [2]. The viscerosomatic reflex can be recognized by the presence of TART [2]. This last condition can cause painful symptoms, traveling from the viscera towards DRG and dorsal horn and contacting the interneurons of the same medullary lamina of entry. The visceral information that enters the spinal cord will create synapses with the alpha motoneurons, and autonomic and somatic neurons (always at the same spinal segmental level); from these contacts, the viscerosomatic reflexes will start [2]. This constant neural mechanism could induce sensitization of the medullary (and then central) area. The presence of a viscerosomatic reflex involves one or more segments, as well as the ipsilateral area of the viscus, with tissue characteristics of sympathetic hyperactivation [2]. Within this vision, we find the theory of the facilitated segment.

An important difference between a somatosomatic reflex and a somatovisceral reflex is the fact that in the latter, there will be a more evident limitation of the joint excursion, as well as altered motor patterns [2]. The somatovisceral reflex will involve the innervation of a viscus of the same specific facilitated segment. Furthermore, this reflex will very often be concomitant with a viscerovisceral reflex, always with the same logic of the facilitated segment [2]. The viscerovisceral reflex is defined as “localized visceral stimuli producing patterns of reflex response in segmentally related visceral structures” [2]. A somatic or visceral dysfunction will always lead to an alteration of all the connected structures, such as blood and lymphatic vessels, bone tissue, and the meningeal component itself [13].

The theory essentially describes a spinal facilitation, probably due to aberrant afferent or efferent activation patterns and return, temporarily or chronically altering the electrical and metabolic environment of synaptic contact, which, once established, may also be self-generating. Should the osteopath follow the facilitated segment theory as a starting point for deciding on the possible therapeutic path?

Visceral afferents

Visceral afferents that derive from the viscera, glands, and vessels are not part of the autonomic nervous system but of the visceral nervous system, even if they run with the sympathetic nervous system [14]. The cell bodies that signal and transport visceral information are pseudo-unipolar cells (T-shaped junction), found within each ganglion of each dorsal root, with a morphology that puts central and peripheral pathways in contact. The structures that record visceral variations can be specialized corpuscles (Pacinian-like) or free endings. The visceral afferent fibers run alongside the sympathetic pathways until they reach their own ganglion of competence (thorax, abdomen, and part of the pelvis), while the cranial and sacral areas derive from parasympathetic pathways [14]. The visceral afferent pathways of the abdomen and thorax, through the splanchnic nerves, enter the trunk of the sympathetic system (without synapses) and the dorsal root, thanks to the grey communicating branches, while the parasympathetic pathways follow the efferent vagal or pelvic splanchnic systems [14].

Once they enter the spinal cord, they mainly travel towards lamina I and V of the medullary dorsal horn, involving several structures, such as the intermediolateral cell column, spinosolitary and spinoparabrachial tracts (interoception and visceronociception), spinotrigeminal tract, spinothalamic tract, Lissauer's tract [15-18]. Visceral afferents represent approximately 10% of the total afferents reaching the spinal cord [19]. There is a close convergence between somatic and visceral information, and this anatomical relationship could explain viscerosomatic referred pain [19]. Likewise, there seems to be a convergence of visceral afferents, probably creating the viscerovisceral symptom [14]. At the cranial level, visceral information reaches the nodose ganglia, the glossopharyngeal ganglia, and facial ganglia [20].

Somatic afferents

The human body has 43 input segments for somatic afferents, counting the cranial and peripheral nerves. At the cranial level, somatic afferents travel to the trigeminal ganglion and the spinal trigeminal nucleus, while peripheral afferents involve the medullary area between lamina I and lamina V, entering from the dorsal horns of the spinal cord. The route to the central nervous system for peripheral somatic afferents will use different columns, such as the dorsal column medial lemniscus, dorsal spinocerebellar tract, and spinocuneocerebellar tract [20]. Afferents are simplistically divided into different fiber subtypes, but we still know little about their actual function and characteristics. These afferents respond to multiple stimuli, thermal, position, pain, touch and being touched, movement, itch, stretching, and pressure [20].

The dorsal medullary area has a somatotopic organization, with medial afferents for the upper and lower limbs, and lateral afferents for more neighboring anatomical areas. Lamina I and II receive nociceptive peptidergic and nonpeptidergic endings, neurons that respond to low-threshold C-type receptors; lamina II-III receive information from low-threshold somatic A-delta receptors; and afferents terminating in lamina III-V carry information from A-beta receptors [20]. Most afferents enter the DRG before entering the medulla. Not all nociceptive and purinergic somatic information enters the corresponding DRG; moreover, many A-beta afferents branch before entering the medulla, giving a cranial branch and a caudal branch, involving multiple medullary levels [20].

Visceral efferents

The parasympathetic efferent system consists of four cranial nerves (III, VII, IX, X); in particular, the general visceral efferent vagal fibers synapse with the autonomic ganglia of the thorax and abdominal area, involving all the viscera of the body, creating synaptic contacts with the post-ganglionic cell bodies [21]. The latter emanate branches to intervene in visceral function, and smooth and glandular muscles. The special vagal visceral efferent fibers innervate the muscle fibers of the cervicothoracic area for the function of speech and swallowing; furthermore, these fibers innervate a portion of the diaphragm muscle (Low's muscle and transverse intertendinous muscle) for the correct relationship between breathing and passage of the bolus [21,22].

Visceral efferents also derive from the sympathetic system (preganglionic fibers and then autonomic ganglia), with fibers that branch out and orient themselves toward target organs (postganglionic neurons). Preganglionic fibers are found in the lateral horn of the spinal cord (T1-L3), leaving the descending pathway through the ventral area of the spinal nerve roots, connecting to them via white branches (visceral efferents) and gray branches (visceral afferents); from here, the fibers arrive in the ganglia [23].

We can find 15 white branches ipsilaterally, while each branch of the spinal nerves receives a grey branch [23]. At the sacral level, the parasympathetic efferents are found in the spinal cord at the level of the lateral medullary horns, then merging with the dorsal and ventral sacral roots (sacral nerves); the pelvic ganglia that will form will be mixed (sympathetic, parasympathetic, and enteric fibers) [23]. The autonomic ganglia, compared to the somatic spinal root ganglia, create numerous synapses, connecting the entire sympathetic and parasympathetic system in an indistinct network.

Afferent/efferent complexity: neurobiochemistry

Tyrosine hydroxylase is an enzymatic catalyst capable of stimulating the synthesis of L-tyrosine to L-3,4-dihydroxyphenylalanine (levo-DOPA), starting from the non-essential amino acid tyrosine (starting from the essential amino acid phenylalanine); from this reaction, the catecholamine dopamine is formed, which is found in large percentages in the pathways of the sympathetic system [24]. It should be noted that dopamine is present in a significant percentage, as it is the precursor to the formation of norepinephrine. The vagus nerve contains different nerve transmission pathways, such as efferent fibers for the viscera, somatic fibers, general afferent fibers of the somato-viscero-sensory type, and special afferent viscerosensory fibers. The vagus nerve also contains catecholaminergic fibers, which are capable of synthesizing dopamine (and noradrenaline); dopamine is a typical neurotransmitter of the sympathetic system. The cranial, cervical, and thoracic vagus nerves contain sympathetic fibers, with a percentage of presence representing less than 10%, with high subjective variability and right and left side diversity [25,26]. In the path of the parasympathetic fibers, from an anatomical point of view, it is evident that they often undergo a fusion between them, and through branches that derive from the same fibers, a neighboring and distant communication is created between them [27]. In animal models, the vagus nerves in the abdomen merge with the celiac ganglion (right and left), which interface with the enteric system [28].

Catecholaminergic cell bodies are found not only in ganglia but also in ventral and dorsal spinal roots [29]. The rami communicans (white and gray) can share the anatomical structure (epineurium), becoming for a short distance a single branch and confusing the informational origin (afference and efference) [29]. The white rami communicans possess not only efferent myelinated fibers but also afferent myelinated fibers; this organization makes these branches both efferent and afferent [29]. Likewise, the gray rami communicans can have many myelinated fibers, further mixing their function [29]. The neural function that appears specific to a given neuron or anatomical area can change based on the connections it receives or produces [29]. The spinal cord is not only a passageway for afferent and efferent information to and from the central nervous system but can also act independently from the intervention of the brain and adapt autonomously, creating rhythmic neural circuits, as a non-hierarchical but heterarchical organization [30]. Within the spinal cord, there are neural areas known as central pattern generators (CPGs). These spinal areas can rhythmically generate nerve impulses for peripheral muscle contraction (locomotion) and some basic functions (respiration, swallowing, chewing) through biochemical oscillations (sodium, calcium, N-methyl-D-aspartic acid, potassium), which are able to produce electrical activity [22,31]. These electrical currents can depolarize neurons that are already physically interconnected and create a CPG; this mechanism occurs without central neural intervention [31]. A stimulus that excites a single neuron can then lead to a spread of activity through the surrounding glia [20,32]. We can state that “the spinal cord is not a simple reflexive machine” [30]. Cells within the spinal cord are not based on unalterable patterns but can change shape and function depending on the chemical and electrical stimulus that arrives, in a time-dependent manner [30]. Afferents can change function, independently of central nervous system control; these mechanisms of plasticity are reminiscent of the central neural mechanisms on which learning and memory are based [30].

New branches or neurites can grow if there is a constant electrical/chemical stimulus; this stimulus increases the intracellular calcium oscillation, which leads to the peripheral production of brain-derived neurotrophic factor (BDNF) at the DRG level. BDNF transcription is mediated by the phosphorylation of cyclic adenosine monophosphate (cAMP) response element-binding protein (CREB), thanks to the presence of a protein contained in presynaptic vesicles such as synapsin I [33]. BDNF travels in retrograde mode to the spinal cord, changing the neural structures. This mechanism also occurs after a long time (weeks) from the interruption of the stimulus (memory and learning) [30]. Afferents can become efferents, carrying biochemical information towards the peripheral termination, for example, towards a viscus, altering its function [34]. Therefore, to maintain a certain non-physiological pattern, efferential intervention is not necessary, canceling the cause-effect relationship as in somatic and visceral reflexes [30].

In the spinal cord and central nervous system, to keep a stimulus active, for example, for movement, the neural bodies are not necessarily fully activated (for example, motor neurons), but an apparently chaotic neural network that varies its electromagnetic oscillation (rhythmogenic properties) is sufficient, managing to produce rhythmic muscular movements, involving or not involving motor neurons. This happens independently of the specificity of the neuron, that is, not linked to its specific function [35,36]. A slight release of glutamate/glycine from oscillating neurons (balanced sequence generator), stimulated by information arriving from the periphery or the central nervous system, is sufficient to create adequate amounts of neurotransmitters to activate/deactivate specific neurons. No one can know, therefore, where the symptom will arise, undermining the concept of somatic or visceral reflex.

Afferent/efferent complexity: distribution of nociceptive information

Another complexity of the spinal cord concerns the distribution of nociceptive information. The distributed nociceptive system is a new model born from recent research.

In the spinal cord, two types of neurons that respond to nociceptive information coexist: nociceptive specific neurons and wide dynamic range neurons. The former are activated by real nociceptive information, while the latter group of neurons is activated not only by nociceptive stimuli but also by non-nociceptive information [37]. Classically, the serial view of informational pathways is a set of spinal segments that are activated following a concept of hierarchical succession until reaching the central nervous system [38]. Furthermore, the spinal dorsal horns are considered as the site of sensory processes, while the ventral horns are considered as the site for the procession of motor data; laminae I and V have been considered as preferential ascending pathways to transport nociceptive information ipsilaterally [38]. The reality of living is more complex. The nociceptive spinal information coming from the periphery (soma and viscera) travels through several laminae (I, V, VI, VII, X) and is distributed in both the dorsal and ventral horns [38]. Furthermore, the ascending pathways activated by the entry into the spinal cord activate not only the ipsilateral segment but also the contralateral one with equal intensity; the spinal activity is not only caudo-rostral but also ipsilateral and contralateral [38,39]. When the nociceptive impulse arrives, several spinal segments are involved (from 3 to 8), involving neurons caudal to the input of the information, cranial, ipsilateral and parallel; not only the primary afferents but also intermedullary proprioceptive pathways are involved [38]. Probably, when these nociceptive signals reach the central nervous system, activating ipsilateral areas with respect to the afferent input and contralateral brain areas with respect to the spinal cord input of the stimulus, they allow the nervous system to process a better response [38,40]. In the human model, it is not a rule that the spinal segment involved by nociceptive afferents respond in a specular manner, that is, the efferent response may arise from other spinal level [41]. The same efferent spinal roots anastomose with the communicating branches, creating dermatomal tissue alterations that do not reflect the classic vision of "1 + 1 = 2" [42]. The afferents that enter the spinal cord, both nociceptive and related to all the information that concerns interoception (viscera, fat, skin, and others), follow pathways involving many tracts of the spinal cord at the same time [43]. This informational network makes the origin of the stimulus indistinguishable; what counts is the symptom (from which to start) and the palpation (on which to work osteopathically). The tables that report the visceral innervation and the relative spinal segment are only one point of view if used in a clinical setting [42-44].

Afferent/efferent complexity: metabolic, endocrine, and systemic immune aspects

We must also consider the metabolic, endocrine, and systemic immune aspects, which are able to alter the afferent/efferent response, independently of the medullary segment. To give some examples, in animal models, it has been shown that the level of estrogen influences the behavior of the pelvic musculature, as well as the smooth muscles of the pelvic viscera (urethra and bladder), independently of the idea of reflexes present in the facilitated segment hypothesis [45]. Similarly, an altered level of thyroid hormones (hypothyroidism) negatively influences the function of the striated muscles of the pelvic floor and the smooth muscles of the pelvic viscera (urethra and bladder) [46].

A low level of systemic inflammation or meta-inflammation increases the circulating levels of pro-inflammatory substances (C-reactive proteins, interleukin-1β, interleukin-6, interleukin-17, tumor necrosis factor-alpha), stimulating the infiltration of macrophages and T lymphocytes in different tissues, peripheral and central, causing multiple symptoms not referable to specific spinal reflexes [47]. We should imagine the spinal cord as the site of not only electrical but also biochemical activities, which, in a three-dimensional context, are always evolving (synaptogenesis). In the presence of a neural contact, for example, for the formation of filopodia, several molecules that promote adhesion are activated (synaptic cell adhesion molecules, neuroligins), and they are the same ones that help presynaptic specialization. Neuroligins located in the post-synaptic membrane help consolidate the contact through the bond they provide to presynaptic neurexins (cell adhesion proteins), organizing the synaptic structure and function [48]. If the need to form new neural patterns ceases or decreases, other mechanisms come into play to disassemble the synapse, such as transforming growth factor-β and the activation of various metabolic and biochemical pathways, such as small Rho GTPase family of proteins/ rho-associated kinases that destructure the synaptic organization [49].

There are other strategies by which a neuron can communicate with another cell body in the spinal cord, independently of the creation of synapses. These mechanisms make the concept of reflexes difficult to use in the clinic.

Every cell in the human body, including neurons, can influence the genetic response of another neuron through the synthesis of deoxyribonucleic acid (DNA) and then the delivery of micro-ribonucleic acids (miRNAs); these small non-coding RNAs travel via extracellular vesicles [50]. MicroRNAs are fundamental for cell function, as they allow continuous genetic updating; it is the genetic expression of each cell that determines function. Specifically, these small molecules repress post-transcriptional genetic expression [51]. The production of these miRNAs determines the behavior and structure of synapses, and of the neural body, influencing their contribution within the nervous system [52]. MicroRNAs travel not only from cell to cell in the same tissue but also from distant tissues to non-neighboring cells, making spinal cord function and responses very difficult to capture in a single view.

Another tool that the cell can use to influence neighboring cells is the creation of tunneling nanotubes (TNTs). These structures are protrusions made of proteins such as actin and tubulin, capable of transporting numerous biochemical and electrical substances over long distances, without the creation of synapses and directly into the cell cytoplasm [53]. TNTs are non-permanent formations and can reform several times [50]. Other similar structures which only bind to the membrane (and not directly into the cytoplasm) are cytonemes. These are filaments or microtubules consisting mainly of actin cytoskeleton. These membrane protrusions are similar to filopodia, which come into contact with another cell membrane for the transport of numerous biochemical substances. These connections can apparently involve non-participating cells in a precise neural pattern, stimulating morphogenesis and influencing the receptor field [54].

Clinical implications

The notions described above do not fit well into the facilitated segment model, but they can be useful to understand clinical pictures that are not always easy to frame.

Referred pain does not always reflect the topography of innervation (neuromeric field), as there is not always a neural convergence in the spinal cord [55]. The symptom that is referred by the patient can arise from distant and apparently unconnected areas, and the reasons for this are not yet fully understood [56]. The idea of the facilitated segment is a vision that reduces the human body as sections and not as a unit; the human body should always be thought of as a unit, regardless of the area of the symptom [57].

The phrenic nerve (spinal nerve) can carry both efferents and somatic and visceral afferents (from the pericardium, stomach, Glisson's capsule, liver, gall bladder, parietal peritoneum), going beyond the mere concept of the facilitated segment [58]. The nerve contains different neural components: motor, sensory, and orthosympathetic fibers. Dysfunctions of visceral areas innervated by the phrenic nerve can send different afferents, which, in the long term, could cause somatic, visceral, and emotional status disorders [59]. A phrenic dysfunction, for example, due to anomalous traction of the common bile duct, can put the peritoneum covering the gallbladder under constant tension; this could create a non-physiological mechano-metabolic environment, which is sensed by the phrenic nerve. Non-physiological afferents carried by the phrenic are carried towards the central nervous system, passing through all the phrenic medullary areas and involving non-phrenic spinal fibers, with which it anastomoses. Chronic non-physiological inputs carried by the subdiaphragmatic phrenic nerve (for example, abnormal tension in its structure) may manifest as a paresthesia in the ipsilateral little finger. All anastomoses of the phrenic nerve (as with all other nerves) represent passages of electro biochemical signals. If the phrenic nerve carries non-physiological inputs, these signals will involve all anastomoses along its path. It is possible that a neural area less able to tolerate these inputs over time could create a dysfunctional neural environment. In this case, the subclavian nerve may be less able to handle inputs from the phrenic nerve, causing an alteration in its electrical activity (increase); this mechanism could induce constant contractions (spasms) of the subclavius muscle. A spasm of the subclavius muscle due to constant phrenic interference on the subclavian nerve could cause a chronic elevation of the first rib, which compresses the ulnar nerve (due to anatomical topography, it is the first nerve that can be compressed by the first rib). This would create a neurological thoracic outlet syndrome but starting from the common bile duct [60].

Retrograde axonal transport, for example, from peripheral tissues to the presynaptic space and the synapse, is mediated by proteins such as dyneins and kinesins, which transport multiple substances (lipids, proteins, organelles, RNA, neurotrophic factors) to the neural body of origin [60,61]. The nerve transports (bidirectionally) many substances for the maintenance or change of the form/function of the neural body [62,63]. An inflammatory condition due to multiple causes stimulates the production of pro-inflammatory cytokines and other structures from the tissues, including the retrograde transport of voltage-gated sodium (NaV) channels, in particular subtype NaV 1.7. NaV 1.7 is transported to the neural membrane, stimulating a series of biochemical and electrical cascades, decreasing the activation threshold of the neurons; This mechanism will lead to a greater sensitization of the neuron to inflammatory substances, which will stimulate nociception and/or convey further non-physiological information towards the central nervous system [64]. In animal models, a colorectal distension can stimulate spinal neurons that are outside a specific segment of afferent input, such as in the trigeminal and cervical area; this leads to a greater nociceptive sensitivity on the skin of the cervical and facial area [65]. Spinal cord neural convergence does not always cause segmental symptoms, but also heterosegmental ones, putting the facilitated segment theory in difficulty [56]. Visceral disorders involving the vagus nerve can have repercussions in the cervical area, up to the trigeminal spinal nucleus, and cause symptoms such as headache or chronic cervical pain [66]. Irritable bowel syndrome could probably be a source of chronic nociceptive afferents, which, by traveling along the spinotrigeminal pathway, could induce somatic symptoms distant from the visceral area of onset [67].

An esophageal disorder (gastroesophageal reflux disease, heartburn) can induce migraine through stimulation and non-physiological retrograde transport of multiple substances (calcitonin gene-related peptide, substance P, various cytokines and chemokines) by visceral afferents. These substances are transported in a retrograde manner until they involve the trigeminal system as the destination [68-70]. Calcitonin gene-related peptide increases neural sensitivity and influences the perception of pain towards supraspinal areas through the activation of different receptors (receptor activity-modifying protein 1, calcitonin receptor-like receptor, amylin 1 receptor); substance P through the activation of specific receptors (neurokinin-1) modulates the perception of pain from the spinal cord towards supraspinal centers [71,72].

The presence of a hypertrophic scar (HS) or a keloid can be the source of symptoms that are not easily classifiable in clinical terms. These scar formations possess a greater number of mechanically activated cation channels, particularly Piezo1, which is extremely sensitive to skin tension changes, such as stretching; this is found in the epidermis (as well as in the peripheral nervous system and medulla), with melanocytes and Merkel cells [73-75]. Its activation can induce the production of nociceptive and inflammatory information, which are emphasized both by retrograde transport in the medulla (substance P, calcitonin gene-related peptide, histamine) and by the activation of these channels present in the meninges by the same inflammatory and nociceptive substances transported [74,76]. Other neural receptors are activated in the medullary horns to carry nociceptive information to multiple medullary levels, such as N-methyl-D-aspartate, gamma-aminobutyric acid-alpha, α-amino-3-hydroxy-5-methyl-4-isoxazolepropionic acid, and voltage-gated calcium channels [76]. A chronic skin alteration, regardless of the site of the damaged skin, can be a source of migraine episodes [77]. Probably, by transporting a constant amount of neuroinflammatory substances towards the spinal trigeminal nucleus and/or towards the Gasser's ganglion, pain phenomena can be triggered, independently of medullary reflex arcs.

The presence of chronic overlapping pain conditions (COPCs) does not correlate with the presence of the facilitated segment hypothesis. COPCs include chronic syndromes in which there is never a single symptom in each body region but multiple concomitant symptoms [78]. Urologic chronic pelvic pain syndrome (UCPPS) often presents with burning mouth syndrome (BMS). Probably, all nociceptive biochemical substances (pro-inflammatory cytokines) transported in retrograde mode from somatic and/or visceral structures entering the spinal cord could activate different nociceptive neural receptors, such as formyl peptide receptor type 2 (FPR2/ALX) [79]. The latter is present throughout the spinal and central system. Therefore, a disorder from the pelvic area could negatively influence the lingual innervation via spinal cord afferents to the supraspinal areas.

The neurological model in the osteopathic field is summarized as follows: “The neurological model considers how autonomic equilibrium, neural reflex activity, segment facilitation, afferent nerve signals, and nociception contribute to injury and disease” [80]. Probably, it would be better to question the concept of the facilitated segment more, considering the numerous neurophysiological information in the literature. Furthermore, it would probably be better to speak of a bioelectrical model since all nervous structures, central and peripheral, are expressed with electrical and biological actions. This last reflection would better reflect the neurophysiological reality that we know, considering, for example, the spinal cord as an organ [81,82].

Based on this theory, the osteopath directs treatment between the facilitated spinal segment and the corresponding reflex area. In reality, the symptom does not necessarily follow the presumed spinal segmental level cited in the textbooks. This means that the osteopathic approach, which relies on spinal reflexes in the neurological model, must necessarily change. Not only is further research needed to better define the osteopathic approach within the neurological model, but also modern osteopaths should likely rely on palpation (always aided by instrumental and validated tests) to identify the most dysfunctional area and guide their treatment. This should be done independently of reflex theory, i.e., leaving pre-existing reasoning patterns that could limit the desired clinical outcome.

## Conclusions

Several scholars and researchers in osteopathic medicine laid the foundations for trying to understand symptomatic behavior through the concept of the somatic and/or visceral reflex arc and inserting it into a neurological model. Segmental spinal facilitation is seen as a chronic irritation of the spinal cord causing dysfunctions related to the afferent/efferent area of the specific spinal segment, with peripheral and central negative influences. The spinal cord is an organ capable of not only transferring ascending and descending information but also processing and modulating such information. Like the entire nervous system, the spinal cord area is rich in biochemical substances imbibed in an electrical network; determining how this area will respond in the presence of afferent and efferent stimulation is not always simple, as the spinal organ is not a scheme but an organism. Based on this theory, the osteopath directs treatment between the facilitated spinal segment and the corresponding reflex area. In reality, the symptom does not necessarily follow the presumed spinal segmental level cited in the textbooks. This means that the osteopathic approach, which relies on spinal reflexes in the neurological model, must necessarily change. The article briefly reviewed some concepts of neurophysiology to raise new reflections on whether or not to consider the hypothesis of the facilitated segment and to have a different vision in clinical pictures that are not always easy to frame.
